# Chromatin and Epigenetic Rearrangements in Embryonic Stem Cell Fate Transitions

**DOI:** 10.3389/fcell.2021.637309

**Published:** 2021-02-18

**Authors:** Li Sun, Xiuling Fu, Gang Ma, Andrew P. Hutchins

**Affiliations:** Department of Biology, Southern University of Science and Technology, Shenzhen, China

**Keywords:** embryonic stem cells, naïve, primed, epigenetic, transposable elements, reprogramming

## Abstract

A major event in embryonic development is the rearrangement of epigenetic information as the somatic genome is reprogrammed for a new round of organismal development. Epigenetic data are held in chemical modifications on DNA and histones, and there are dramatic and dynamic changes in these marks during embryogenesis. However, the mechanisms behind this intricate process and how it is regulating and responding to embryonic development remain unclear. As embryos develop from totipotency to pluripotency, they pass through several distinct stages that can be captured permanently or transiently *in vitro*. Pluripotent naïve cells resemble the early epiblast, primed cells resemble the late epiblast, and blastomere-like cells have been isolated, although fully totipotent cells remain elusive. Experiments using these *in vitro* model systems have led to insights into chromatin changes in embryonic development, which has informed exploration of pre-implantation embryos. Intriguingly, human and mouse cells rely on different signaling and epigenetic pathways, and it remains a mystery why this variation exists. In this review, we will summarize the chromatin rearrangements in early embryonic development, drawing from genomic data from *in vitro* cell lines, and human and mouse embryos.

## Introduction

Each cell contains the same DNA that is interpreted to provide specialized cell function, yet the interpretation of the DNA code is cell type-specific, and epigenetic barriers exist that impair and permit cell type conversions. Cell type conversions are surprisingly rare in the adult organism, outside some stem cells, which retain limited ability to derive various progeny (Avgustinova and Benitah, 2016). Despite the importance of understanding cell type control for regenerative therapies, exactly how this process is controlled remains surprisingly elusive (Hutchins et al., 2017). Epigenetic control mechanisms are a major contributor to this process; however, there are a wide array of overlapping and competing mechanisms, particularly histone and DNA chemical modifications (Ohbo and Tomizawa, 2015). Epigenetic modifications form barriers that can be permissive for some cell type transitions but intolerant for others. These epigenetic barriers can resist all cell type conversions but can also act as bidirectional valves, guiding cells toward a differentiated cell type but then blocking reversion to progenitor cells and locking cells into a fixed cell type (Arabaci et al., 2020). Epigenetic control is mediated by DNA-binding proteins, particularly transcription factors (TFs) (Fu et al., 2017), that recruit epigenetic enzymes to regulate chemical modifications on DNA and histones and reshape or remodel chromatin structure. This process both responds to cell type changes and controls them and is critical in normal developmental processes. During embryonic development of a new organism, epigenetic information is reset (Guo et al., 2014; Xia and Xie, 2020), and many epigenetic rearrangements occur during early pre-implantation development and accompany, and in some instances drive, developmental changes. Research to understand the epigenetic reconfigurations in embryonic development has been intense, as understanding epigenetic control could give fine-grained control of cell type states, which would be useful for a wide range of medical applications, from regeneration (Avgustinova and Benitah, 2016), and cell replacement therapy, to understanding how cell type control is disrupted in cancerous cells (Meacham and Morrison, 2013). In this review, we will discuss epigenetic control of early embryonic stages, using work from embryos, *in vitro* mimics of embryonic cells, and cell type conversion systems.

## NaÏVe and Primed Cells in Mice

**FIGURE 1:**
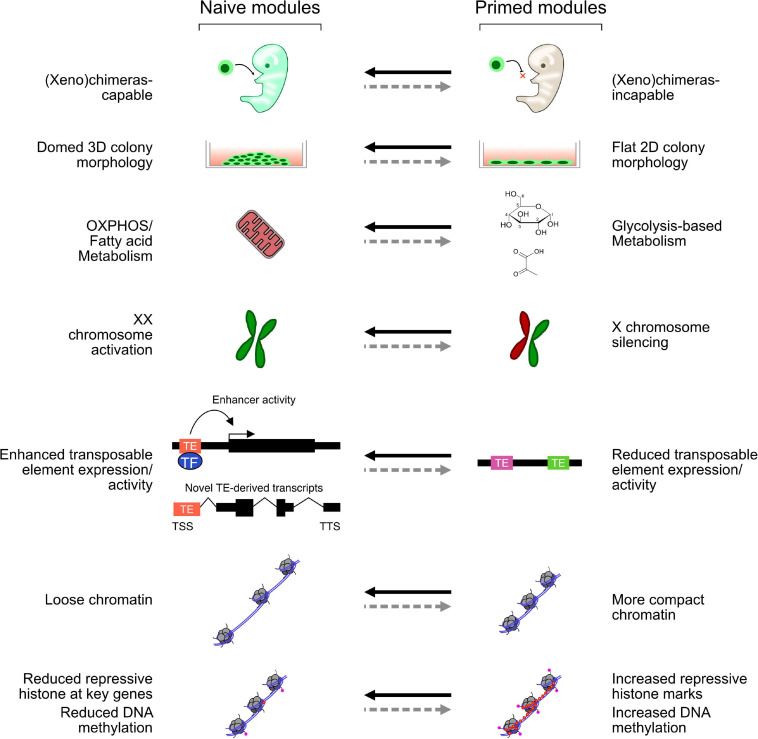
Selected modules representing key features of naïve and primed cells. Each module can be switched on and off, perhaps independently, in naïve and primed mouse and human embryonic stem cells (hESCs). While mouse ESCs (mESCs) activate all these modules, different naïve-like hESCs may only activate some aspects.

Amongst the first *in vitro*-derived embryonic cells were the human and mouse embryonal carcinoma (EC) cell lines, which can be maintained *in vitro* but are derived from teratocarcinomas and have multiple mutations and abnormal karyotypes (Martin, 1980). Several EC lines were derived, and while each line has a set of common embryonic features, they also have line-specific effects, such as restricted differentiation potential and different culture requirements (Andrews, 1988; Alonso et al., 1991). When ECs are injected into a mouse blastocyst, they can contribute to development but often lead to teratomas in the adult mice. Fully viable EC-derived chimeras have been reported (Hanaoka et al., 1987); however, considering what we now know about the rapid growth of normal, untransformed mouse embryonic stem cells (mESCs), it is not inconceivable that those EC cultures harbored small numbers of mESCs. Compared with ECs, mESCs are untransformed, contribute to chimeras at high frequency without generating teratomas in the adult, and can be grown *in vitro* in defined conditions indefinitely. *In vitro*, mESCs can be differentiated to all three germ lineages and have become a powerful model of the early stages of embryonic development (Rossant, 2011). mESCs are thought to most closely resemble the early epiblast (Boroviak et al., 2014). They express marker genes specific for the epiblast; and in female mESCs, both X chromosomes are active; and silencing is required to exit the pluripotent state (Schulz et al., 2014). mESCs were first derived in 1981 (Evans and Kaufman, 1981), but it was not until 1998 that human ESCs (hESCs) were reported (Thomson et al., 1998). However, there are several morphological and molecular differences between mESCs and hESCs: mESC colonies are domed, hESCs are flat, hESCs rely on glycolysis, and mESCs oxidate phosphorylation (Zhou et al., 2012; Sperber et al., 2015). There are also major differences in the ectopic signaling pathways that are required: mESCs rely on bone morphogenic protein 4 (BMP4) and leukemia inhibitory factor (LIF) signaling (Ying et al., 2003); however, hESCs rely on Activin A and fibroblast growth factors (FGFs) (Beattie et al., 2005); and not only is BMP signaling not required for hESCs, but inhibition of BMP signaling is even beneficial (Xu et al., 2005). Application of the hESC growth medium to mouse cells led to the isolation of epiblast stem cells (EpiSCs). These cells were derived from E6.5 pre-gastrulating embryos and are quite different in morphology, gene expression, and culture conditions than mESCs (Brons et al., 2007; Tesar et al., 2007). EpiSCs, instead, more closely resemble hESCs, and the pre-gastrulating epiblast, a later developmental stage than mESCs. To explain the properties of mESCs and EpiSCs, a two-phase model of embryonic development was proposed, consisting of a “naïve” (mESC) state, which is closer to the early epiblast, and a “primed” (EpiSC) state, which is closer to the late pre-gastrulating epiblast. Other cell types exist on a continuum between these two conditions and, sometimes, outside of this classification (Nichols and Smith, 2009). Several features distinguish the naïve and primed states (Figure 1). Primed and naïve cells have a distinctive morphology: primed cells are flat and more two-dimensional, while naïve cells have a domed shape and are more three-dimensional (3D). Primed cells are more glycolytic, while naïve cells rely more on oxidative phosphorylation (Zhou et al., 2012). In females, the X chromosomes are active in naïve but silent in primed cells. Finally, in the naïve state, the chromatin tends to be looser and, overall, less repressive, which enables more transposable element (TE) expression.

### The Naïve and Ground States in Mouse Embryonic Stem Cells

Typical mESCs are grown in serum supplemented with the cytokine LIF (Smith et al., 1988; Williams et al., 1988) and adopt a “naïve” state. mESCs can also be supported in medium referred to as ground state or “2iLIF” medium, which consists of PD0325901 (a MEK inhibitor), CHIR99021 (a GSK3 inhibitor), and LIF (Ying et al., 2008). Intriguingly, growth in 2iLIF improves the cells, making them easier to maintain, less heterogeneous, and less prone to spontaneous differentiation (Kolodziejczyk et al., 2015; Takashima et al., 2015). Cells grown in 2iLIF also have an altered chromatin state and reduced levels of repressive marks; for example, H3K27me3 levels are reduced at developmental genes (Marks et al., 2012) and lower levels of other repressive histone modifications (Sim et al., 2017). The change in DNA methylation is more drastic: serum + LIF-grown mESCs have slightly reduced DNA methylation levels, compared with somatic cells, but cells grown in 2iLIF are nearly completely demethylated (Habibi et al., 2013; Leitch et al., 2013). The mechanism appears to be mediated by one of the inhibitors in the 2i cocktail, the MEK inhibitor PD0325901, which indirectly reduces the levels of H3K9me2 and so blocks the recruitment of UHRF1 to chromatin. The loss of UHRF1 then leads to a failure to recruit the methyltransferase maintainer DNMT1, and so DNA is passively demethylated as cells divide (von Meyenn et al., 2016). A complementary mechanism posits a more active process involving MEK inhibitor indirectly stabilizing the histone H3K9me3 demethylase KDM4C (JMJD2C). This leads to demethylation of H3K9me3, the active conversion of 5mC to 5hmC by TET1 hydroxylase, and eventual demethylation (Sim et al., 2017). The global DNA demethylation in naïve cells is reminiscent of a similar DNA demethylation that occurs in early embryonic development between the two-cell (2C) and blastocyst stages (Guo et al., 2014; Smith et al., 2014). However, how close MEK inhibitor-driven DNA demethylation is to embryonic DNA hypomethylation is unclear. Another issue that is complex in 2iLIF-grown cells is that while H3K27me3 at gene promoters is reduced, 2iLIF cells have increased overall H3K27me3 levels and often have increased heterochromatic marks (van Mierlo et al., 2019). This suggests that 2iLIF cells have an overall more repressed chromatin state. One possibility is that the loss of DNA methylation leads to compensatory mechanisms that repress genes and generate heterochromatin by methylating histones. Indeed, H3K27me3 and H3K9me3 compensate for the loss of DNA methylation to repress TEs in mESCs (Walter et al., 2016).

The bromodomain-containing protein BRD4 is an epigenetic reader, which binds to acetylated histones. *Brd4* knockout mice lack an inner cell mass, and when *Brd4* was inhibited or knocked down in naïve cells, it led to differentiation, at least partly due to the loss of *Nanog* expression and other pluripotency genes (Di Micco et al., 2014; Liu et al., 2014; Horne et al., 2015). BRD4 also has a key role in maintaining enhancers, by recruiting CDK9 and the mediator complex (Di Micco et al., 2014). Beyond the direct regulation of pluripotent genes, BRD4 has a complex role in mediating the differences between the 2iLIF ground state and serum + LIF-grown naïve cells, as 2iLIF cells can tolerate the loss of *Brd4*, while serum + LIF cells cannot (Zhang et al., 2020b). The mechanism involves the inhibition of GSK3 in 2iLIF conditions, which leads to stabilization of beta-catenin, and the recruitment of BRD4 and a multimolecular protein complex to pluripotency genes to make 2iLIF-grown mESCs resistant to differentiation (Zhang et al., 2020b). This results in reduced pause-release of RNA polymerase II (polII) and more stable transcription, which helps explain previous observations that 2iLIF-grown cells have more homogenous gene expression than serum + LIF-grown cells (Kolodziejczyk et al., 2015). The mediator complex is a multiprotein complex that is important at integrating signals to activate nearby gene expression by recruiting polII. It has a key role in regulating super-enhancers, which are large regions of DNA with a potent enhancer activity that are important for regulating cell type-specific genes (Whyte et al., 2013). Mediator, in addition to being recruited by BRD4, also has a role in naïve and primed states, as chemical inhibition of two CDKs, CDK8/19, promotes the naïve state in both humans and mice (Lynch et al., 2020). This effect is driven by the hyperactivation of enhancers, as inhibition of CDK8/19 leads to derepression of mediator. BRD4 is not the only bromodomain-containing protein involved in naïve and primed state control. BRD9 was identified as a member of a non-canonical chromatin remodeling BAF complex, and when BRD9 was inhibited, the cells began to acquire aspects of EpiSCs, and, like BRD4, *Brd9* is dispensable in 2iLIF conditions (Gatchalian et al., 2018).

Chromatin inside the cell is tightly packed into successive 3D layers that can be broadly divided into a hierarchy of three organizational features (Rowley and Corces, 2018). The first level is the A and B compartments, which, very roughly, correspond to euchromatin (A compartment) and heterochromatin (B compartment). At the second level, topologically associated domains (TADs) are megabase domains of chromatin that extensively interact within a TAD but weakly between TADs. Finally, at the third level, individual TFs and epigenetic factors form contacts between strands of DNA to form chromatin loops, which are often responsible for bringing distal enhancers together with promoters. mESCs have unique features at all three of these levels, which are suggestive of open and relaxed chromatin. As mESCs are differentiated to neurons, the A compartments decrease and the B compartments increase in interaction frequency, indicating the loss of active chromatin and the acquisition of repressed chromatin as mESCs differentiate (Bonev et al., 2017). In human cells, the situation is similar, and a high-resolution HiC dataset in hESCs and somatic cells showed many A to B compartment switches (Dixon et al., 2015). At the second level, TAD compartment structure strengthened as mESCs differentiated, and TADs containing actively expressed genes interacted weakly, while inactive TADs increased (Bonev et al., 2017). The chromatin state can also influence the 3D genome folding, as knockout of the H3K9me1/2 methyltransferase *Ehmt2* led to reduced TAD boundary strength, although compartments were unaffected (Jiang et al., 2020).

Intriguingly, the 3D structure in developing embryos is initially undefined. From the zygote to the late 2C stage, the TADs and chromatin loops are nearly completely absent, and only compartments on the paternal genome are weakly present (Du et al., 2017; Ke et al., 2017). TADs and chromatin loops reestablish at the eight-cell to the morula stages (Du et al., 2017; Ke et al., 2017). TADs and compartments reform around the same time as zygotic genome activation (ZGA), and there is some evidence that the reestablishment of 3D structure can influence embryonic development. In somatic cell nuclear transfer (SCNT) experiments, the somatic nucleus inside the oocyte briefly retains TADs, which are relaxed at the 2C stage and match normal development. However, the brief window when TADs are erroneously present impairs minor ZGA and embryonic development at the 2C stage, which can be rescued by depleting cohesin to help disrupt TADs in the somatic nucleus (Zhang et al., 2020a). Based on these observations, the totipotent stages of embryonic development (zygote to late eight-cell stage) seem to require relaxed unstructured 3D chromatin. However, it is unclear if this is a necessary feature of totipotency or a consequence of epigenetic reprogramming in early embryogenesis or simply reflects rearrangements in chromatin that are independent of embryonic development. It would be useful to explore these issues in the more experimentally tractable mESCs, and several systems have been explored that lead to dissolution of the 3D genome. With the use of a degron system, the key cohesin complex member RAD21 was deleted in mESCs, leading to the near-complete loss of TADs but a slight strengthening of A/B compartments, and RING1B-mediated polycomb loops persisted (Rhodes et al., 2020). However, depletion of RAD21 in mESCs had surprisingly little effect on gene expression or cell phenotype (Rhodes et al., 2020). CTCF, a major 3D genome organizer, was similarly degraded in mESCs. A/B compartments were unaffected, but TADs and chromatin loops were disrupted; however, once again, the effect on the mESC phenotype was modest, although there was a proliferation defect if CTCF loss persisted (Nora et al., 2017). These results suggest that 3D structure is relatively uncoupled from cell type control, although the precise 3D structure of embryonic cells has not been fully recapitulated in mESCs and hence remains inconclusive.

A remarkable feature of mESCs is their tolerance for the loss of epigenetic regulatory enzymes with relatively few effects (He et al., 2019). For example, components of the polycomb repressor complex 1 and 2 (PRC1 and PRC2) are dispensable for mESCs (Chamberlain et al., 2008). Loss of *Rybp*, a member of an atypical PRC1 complex, also has little to no effect (Li et al., 2017b). Knockdown of *Setdb1*, a H3K9me3 methyltransferase, only predisposes cells to differentiation (Karimi et al., 2011). Co-activators also show the same pattern: *Kmt2d* (MLL2), a H3K4 methyltransferase, is dispensable (Lubitz et al., 2007). Epigenetic factor knockouts often do not substantially impact the pluripotent state, although they may make them more prone to spontaneous differentiation and alter the differentiation direction of the cells, or, as is often the case for epigenetic factor knockouts, lead to embryonic arrest at gastrulation, for example, in the *Kdm1a* (Macfarlan et al., 2011) or *Setdb1* (Dodge et al., 2004) knockouts. A CRISPR/Cas9 screen identified around 40 epigenetic factors that, when knocked out, delayed the differentiation of mESCs, and just two epigenetic factor knockouts promoted mESC differentiation, *Cbx7* and *Sin3b* (Li et al., 2018). As their screen was set up primarily to detect improved or impaired ability to differentiate, knockouts that did not affect differentiation would not be detected. This emphasizes the remarkable ability of mESCs to tolerate the widespread loss of epigenetic regulators. This tolerance may be related to the more open and active chromatin, which appears to be a feature of early embryonic cells (Boskovic et al., 2014; Schlesinger and Meshorer, 2019).

### Transcriptional Control of Primed Mouse Epiblast Stem Cells

Primed EpiSCs are a distinct cell state, compared with mESCs. EpiSCs show both molecular and phenotypic differences, particularly colony morphology and the lack of ability to form chimeras (Figure 1). Despite these phenotypic differences, mESCs and EpiSCs have both shared and distinct transcriptional regulation. The core pluripotent network of OCT4, SOX2, and NANOG are active in both cell types (Weinberger et al., 2016), but they bind to different genomic loci (Buecker et al., 2014; Galonska et al., 2015; Matsuda et al., 2017). The core pluripotency network is coordinated by a different set of TFs in each cell type. For example, ESRRB, NR0B1, ZFP42, and TFCP2L1 are important in mESCs (Festuccia et al., 2012; Hutchins et al., 2013; Adachi et al., 2018; Atlasi et al., 2019), but ZIC2, ZIC3, POU3F1, and OTX2 are key in EpiSCs (Acampora et al., 2013; Matsuda et al., 2017; Yang et al., 2019c). Thus, there is a core gene expression module that is common to mESCs and EpiSCs, and divergent regulatory modules specific to each cell type. Both cell types are centered around SOX2-OCT4, but mESCs use TFs that are expressed in the early blastocyst (e.g., NANOG, ESRRB, and TFCP2L1), and EpiSC-specific TFs tend to be expressed in the gastrulating blastocyst (OTX2 and POU3F1). In addition to transcriptional differences, the chromatin and epigenetic states are also altered between EpiSCs and mESCs, and enhancer usage is dramatically altered between EpiSCs and mESCs. Even though only a few hundred genes change expression between mESCs and EpiSCs, several tens of thousands of enhancer marks are differentially regulated (Factor et al., 2014). Even genes that are expressed in both cell types can utilize different enhancers (Factor et al., 2014). This effect had been seen previously at the *Pou5f1* locus, which has two enhancers, a distal enhancer that is active in preimplantation embryos and mESCs, and a proximal enhancer that is active in the epiblast and EpiSCs (Yeom et al., 1996). Yet the global profiling of chromatin highlighted how widespread this phenomenon is (Factor et al., 2014). This redistribution of enhancers is ultimately driven by changes in the TF activity, which decommission and activate panels of enhancers to control each cell state. A good example is OCT4 and SOX2, and both are expressed in mESCs and EpiSCs but are drastically altered in their binding patterns in the two-cell types (Matsuda et al., 2017). It is most likely that OCT4 and SOX2 binding is altered due to the activity of OTX2 and POU3F1, which becomes the dominant factors in EpiSCs (Matsuda et al., 2017).

### Interconversion Between Mouse Naïve and Primed Cells

Mouse embryonic stem cells and EpiSCs can be interconverted *in vitro*, and while conversion of mESCs to EpiSCs is relatively efficient, the conversion of EpiSCs to ESCs remains inefficient without transgenes or epigenetic modulation (Zhou et al., 2010; Tosolini and Jouneau, 2016; Stuart et al., 2019). This indicates that EpiSCs are developmentally later, as, in general, cells can efficiently differentiate toward their progeny but are resistant to dedifferentiation to their precursor cell type. Epigenetic barriers, particularly unidirectional blocks, appear to permit the conversion of mESCs to EpiSCs but block the reverse. ZFP281 acts as just such a bidirectional valve, as it assists mESC to EpiSC conversion but blocks the reverse (Mayer et al., 2020). The mechanism involves ZFP281 co-binding with EHMT1 to methylate H3K9 and inhibit genes in the early stages of mESC to EpiSC conversion (Mayer et al., 2020), while in the reverse case ZFP281 binds the NuRD co-repressor complex to suppress *Nanog* expression and enable exit from pluripotency (Fidalgo et al., 2012). When mESCs are converted to EpiSCs, there is a global reconfiguring of chromatin (Factor et al., 2014). These properties have made the interconversion of mESCs to EpiSCs a powerful model to understand the epigenetic modulation of cell conversions.

Several factors have been identified that influence the conversion of mESCs to EpiSCs (Table 1). Most are TFs that promote the conversion of EpiSCs to mESCs. These TFs recruit other co-activators and co-repressors to influence the chromatin state, although their direct activity is not always clear, as TFs can often act as both activator and repressor in a context-specific manner. For example, NANOG is mainly an activator but can also work as a repressor (Heurtier et al., 2019). When NANOG binds to DNA with ZFP281, it recruits the NuRD histone deacetylase (HDAC) co-repressor complex (Fidalgo et al., 2012). *Esrrb* is a major requirement to convert EpiSCs to mESCs, as it participates in extensive chromatin remodeling (Adachi et al., 2018). Many naïve-specific enhancers are kept silent in EpiSCs by DNA methylation and inaccessible chromatin. ESRRB opens these naïve-specific enhancers by recruiting the p300 complex, displacing and phasing nucleosomes, and opening closed chromatin, making it accessible for other members of the pluripotency regulatory network, such as OCT4, SOX2, and NANOG, to bind (Adachi et al., 2018).

**TABLE 1:** Selected factors that influence the interconversions of mESCs and EpiSCs.

Molecule(s)	Function	Effect on mESCs, EpiSCs, and their interconversion	References
Brd9/BAF complex	Chromatin remodeler	BRD9, through a non-canonical BAF complex, blocks transition to a primed state	Gatchalian et al., 2018
Epiblastin A	Inhibitor	Inhibits CK1 and induces EpiSC-to-mESC conversion	Illich et al., 2016
Esrrb	Transcription factor	Overexpression sustains LIF-independent self-renewal in the absence of Nanog and reverts EpiSCs to mESCs	Festuccia et al., 2012; Adachi et al., 2018
Hif1a	Transcription factor	HIF-1α activation switches mESC metabolism and pushes cells toward an EpiSC-like state	Zhou et al., 2012
iPStat3	Gp130^*Y*118*F*^ receptor	Artificially induces STAT3 signaling and converts mESCs to EpiSCs	Stuart et al., 2019
Kdm1a/Kmt2d (MLL4)	Histone methylation	Kmt2d (MLL4) is required to exit pluripotency; knockdown of Kdm1a restores the ability of mESCs to convert to EpiSCs	Cao et al., 2018
Kdm6b (JMJD3)	Histone demethylase	Demethylates H3K27me2 or H3K27me3; Kdm6b facilitates a Klf4-driven EpiSC-to-ESC conversion	Huang et al., 2020
Klf2, Klf4	Transcription factor	Overexpression sustains LIF-independent self-renewal and can convert EpiSCs to mESCs	Guo et al., 2009
Kmt2a (MLL1)	Epigenetic inhibitor	H3K4me1 methyltransferase, deletion of Kmt2a (MLL1) impairs mESC differentiation to EpiLCs; MLL1 Inhibition reprograms EpiSCs to mESCs	Zhang et al., 2016
Nanog	Transcription factor	Transient transfection of Nanog mediates reprogramming of mESCs to EpiSCs	Silva et al., 2009
Nr5a1, Nr5a2	Transcription factor	Overexpression reprograms EpiSCs to mESCs	Guo and Smith, 2010
Otx2	Transcription factor	Required to stably establish EpiSCs	Acampora et al., 2013
Prdm14	Epigenetic factor	Overexpression drives EpiLCs to mESC-like cells	Okashita et al., 2016
Sall1	Transcription factor	Promotes reprogramming EpiSCs to mESCs	Yang et al., 2019a
Setdb1	Histone methyltransferase	Methyltransferase for H3K9me3; Setdb1 loss enriches a transient 2C-like state in mESCs	Wu et al., 2020
Smarcad1	SWI/SNF helicase	Knockdown converts mESCs to EpiSC-like cells	Xiao et al., 2017
Tcf3, Etv5, Rbpj	Transcription factors	mESCs lacking Tcf3, Etv5, and Rbpj are trapped in a naïve pluripotent condition and are difficult to differentiate	Kalkan et al., 2019
Tfe3	Transcription factor	Nuclear-localized TFE3 blocks mESCs from differentiating	Betschinger et al., 2013
Wnt, Gsk3b	Transcription factor	Wnt signaling blocks the conversion of mESCs to EpiSCs and maintains mESCs in the naïve state	Ying et al., 2008; ten Berge et al., 2011
Zbtb7a/b	Transcription factor	Knockdown converts EpiSCs to naïve mESCs	Yu et al., 2020b
Zfp281	Transcription factor	Deletion of Zfp281 promotes EpiSCs reprogramming and acts downstream of Ehmt1 (G9a-like protein; a histone methyltransferase for H3K9me1/2)	Mayer et al., 2020
Zfp706	Transcription factor	Deletion of Zfp706 promotes mESC self-renewal and promotes EpiSC reprogramming	Leeb et al., 2014

*mESC, mouse embryonic stem cell; EpiSC, epiblast stem cell; LIF, leukemia inhibitory factor; EpiLC, epiblast-like cell.*

Epigenetic pathways have been directly implicated in the conversion of mESC to EpiSCs. Activatory epigenetic marks are also redistributed between mESCs and EpiSCs (Factor et al., 2014), and the enhancer mark H3K4me1 has been directly implicated in the conversion to mESCs. Inhibition of the histone methyltransferase that catalyzes H3K4me1, MLL1, drives EpiSCs back to a naïve state (Zhang et al., 2016). In addition to H3K4me1/MLL1 inhibition, an inhibitor of the histone H3K4/9 demethylase KDM1A (LSD1) was part of a cocktail of chemicals that could promote the conversion of EpiSCs to mESCs (Zhou et al., 2010), underlining the importance of epigenetic modulation in cell type conversions. Histone citrullination is the post-translational conversion of arginine to citrulline, and it can act as an epigenetic mark, although its functions are not well defined. In naïve mESCs, histone H1 is citrullinated and evicted from chromatin, decondensing chromatin and likely making it more accessible for TF binding (Christophorou et al., 2014). Histone H3 can also be citrullinated, and it can recruit the SWI/SNF chromatin remodeler SMARCAD1 to relax chromatin (Xiao et al., 2017). Knockdown of *Smarcad1* led to H3K9me3 deposition and heterochromatin spreading, and the cells adopted features of EpiSCs (Xiao et al., 2017). This suggests that citrullination assists in the control of heterochromatin and the maintenance of the naïve state. Overall, these observations agree with the idea that naïve cells represent an “unprogrammed” ground state with lower levels of both repressor and enhancer marks and agree with the idea that less overall epigenetic regulation is a feature of naïve mESCs (Schlesinger and Meshorer, 2019).

**FIGURE 2:**
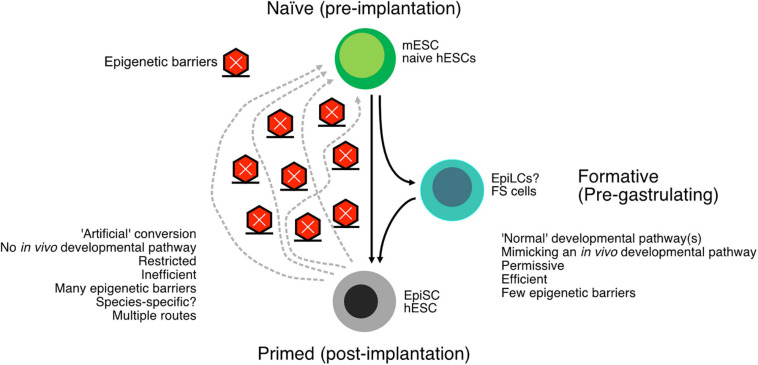
Schematic of the conversion of naïve embryonic stem cells (ESCs) to primed ESCs. The “downstream” pathway follows a normal developmental program and tends to be relatively easy and efficient. The reverse process, reverting primed cells to naïve cells, is arguably an artificial process and consequently more difficult and inefficient. Epigenetic barriers exist between the primed and naïve cells, and there may be more than one way to proceed through these epigenetic barriers. The many epigenetic barriers and the divergent pathways have likely contributed to the difficulty in generating human naïve ESCs. In addition to the naïve and primed states, there is also a formative state that exists intermediate to naïve and primed cells but is capable of primordial germ cell (PGC) formation.

In summary, the naïve and primed states are relatively well described in mouse cells, and it is possible to interconvert the cell types. While conversion from mESCs to EpiSCs is relatively easy, the converse transition is difficult and often inefficient without transgenes. Indeed, there are multiple trajectories to convert EpiSCs back to mESCs, with some mechanisms passing cells through later developmental stages, such as mesoderm-like cells, and other conversion methods pass cells through earlier developmental stages (Stuart et al., 2019). It appears that there is a single main pathway in the differentiation of mESCs to EpiSCs but multiple pathways that EpiSCs must be forced along to revert to mESCs. These observations suggest the existence of epigenetic barriers, probably many, that act to impair the dedifferentiation of EpiSCs to mESCs (Figure 2). Ultimately, the interconversions of mESCs and EpiSCs are a crucial window into epigenetic controls that underlie cell type conversions.

### An Intermediate, Formative Pluripotent State

Work on the naïve state in mice and humans has led to the emergence of a more subtle conception of naïve and primed states. Instead of existing as a binary state, either naïve or primed, there is instead a spectrum of states, some stable and others unstable, that exist between naïve and primed states. A recent proposal posits the existence of a critical intermediate state, the formative state, that exists between naïve and primed mESCs (Smith, 2017). The formative state represents the loss of naïve pluripotency but precedes lineage commitment. One of the first events in the *in vitro* differentiation of mESCs is the loss of the naïve-specific TFs, such as *Tfcp2l1* and *Esrrb*. This matches a similar loss in the early post-implantation epiblast (Boroviak et al., 2014) and precedes lineage priming and acquisition of primed-specific genes. A formative state helps explain some curious phenomena that are not easily reconciled with a binary naïve and primed model. First, deriving primordial germ cells (PGCs) from both mESCs and EpiSCs is inefficient, despite both cell types being on the presumptive developmental path capable of generating PGCs (Hayashi et al., 2011). Second, a third type of *in vitro* epiblast cell, epiblast-like cells (EpiLCs), can give rise to PGCs at relatively high efficiency and can adopt a gene expression profile more reminiscent of E5.0–E6.0 pre-gastrulating blastocysts (Hayashi et al., 2011), which is similar to the timepoint for the specification of PGCs in the embryo (E5.5–E6.25) (Ohinata et al., 2009). This suggests that EpiLCs represent a transient window when PGCs are specified. An interesting aspect of the formative state is the extensive remodeling of chromatin that provides a blank slate for later lineage specification (Smith, 2017). Indeed, EpiLCs have lower levels of repressive histone marks, particularly H3K27me3 and H3K9me2, and have higher levels of bivalent promoters, marked by both H3K4me3 and H3K27me3 (Kurimoto et al., 2015; Yang et al., 2019b). This suggests the EpiLCs are poised for lineage commitment. A comprehensive multi-omic exploration of the conversion of mESCs to EpiLCs revealed a series of waves of gene expression changes that were preceded by widespread chromatin remodeling (Yang et al., 2019b). The conversion to a formative state relies on the expression and activity of *Tcf3*, *Etv5*, and *Rbpj*, as the deletion of all three impairs the differentiation of mESCs to EpiLCs (Kalkan et al., 2019). Of these three factors, their mechanism is unclear, but ETV5 binds to formative state-specific genes and promotes their expression, at least partly through histone acetylation (Kalkan et al., 2019), and all three combine to suppress the naïve pluripotency program.

A disadvantage of EpiLCs is their transient nature, and they cannot be captured *in vitro* like mESCs and EpiSCs. This makes a detailed exploration of the formative state challenging. Recently, two groups reported the isolation of cell lines that fulfill the properties of the formative state but can be maintained in culture (Kinoshita et al., 2020; Yu et al., 2020a). These cells [formative state (FS) and XPSCs] have a gene expression program distinct from mESCs and EpiSCs, yet they can partially contribute to mouse chimeras and can contribute to the germline (Kinoshita et al., 2020) and can generate PGCs *in vitro* (Yu et al., 2020a). Both FS and XPCs show increased bivalent genes, marked by H3K4me3 and H3K27me3 (Kinoshita et al., 2020; Yu et al., 2020a). Overall, in the naïve state, chromatin is open and lacking repressive marks, while in the FS, the cells begin to acquire bivalent chromatin marks that poise the cells for later lineage commitment.

## NaÏVe and Primed States in Humans

Human ESCs were first derived in 1998 from blastocyst-stage embryos (Thomson et al., 1998). hESCs are quite different from mESCs and require Activin A and FGFs, rather than serum/2i and LIF that mESCs need. Such a large difference in growth requirements led to research into why hESCs and mESCs were so different. As described above, mouse EpiSCs could be derived that more closely resemble hESCs, based upon morphology and marker gene expression (Brons et al., 2007; Tesar et al., 2007; Rossant, 2011). However, this prompted a question: If EpiSCs resembled hESCs, is it possible to generate naïve hESCs? Subsequent research began from the basis that mESCs are easiest to derive and stable in ground-state 2iLIF media (Ying et al., 2008), and most naïve hESCs began with this cocktail. However, much as the conversion of EpiSCs to mESCs is challenging, likely due to multiple routes and potent epigenetic barriers (Figure 2), the transition of human primed to naïve pluripotency has also been challenging. The first reported naïve conditions for hESCs involved the transfection of ectopic *OCT4*, *KLF2*, and *KLF4*, along with 2iLIF (Hanna et al., 2010). Since then, there has been a veritable explosion of competing protocols for naïve hESCs, including many that do not require transgenes (Yilmaz and Benvenisty, 2019). However, the situation remains complex, and there is considerable argument about the nature of the putative naïve hESCs.

### Transcriptional and Epigenetic Control of Human Naïve and Primed Cells

The naïve and primed states are well described in mice, but in humans, the situation remains complex (Davidson et al., 2015). Research using human cells has led to the development of a different model of naïve and primed states, which suggests instead of distinct naïve and primed cell states; there are instead modules that can be switched on and off relatively independently of one another (Figure 1). In this model, naïve ESCs switch on a set of modules, while primed ESCs switch on a different set. This view has emerged due to the difficulty in establishing a human version of the complete mouse naïve state and the existence of naïve-like cells that only partially fulfill the naïve criteria. Human naïve ESCs cannot be derived using only 2iLIF; instead, a large number of protocols have been developed that give rise to cells that mimic several aspects of mouse naïve cells (Chan et al., 2013; Gafni et al., 2013; Wang et al., 2014; Ware et al., 2014; Duggal et al., 2015; Carter et al., 2016; Theunissen et al., 2016). However, the competing protocols have distinct transcriptional profiles, cell surface markers, and epigenome states (Yang et al., 2019b), and no comprehensive model has emerged concerning the mechanisms controlling these states in humans. Indeed, just like the conversion of primed EpiSCs to naïve mESCs, there appear to be multiple routes from primed hESCs to naïve pluripotency (Duggal et al., 2015), and potent epigenetic barriers resist the transition. Both naïve and primed hESCs are regulated through several core TFs, for example, SOX2 and OCT4, which are common to both naïve and primed hESCs in mice and humans. However, the human cells have gene regulatory networks that utilize KLF5, KLF7, TFCP2L1, FOXR1, ZIC2, and TFAP2C, for the naïve state; and OTX2 and SALL2 for the primed state (Takashima et al., 2014; Weinberger et al., 2016; Pastor et al., 2018). Some of these are active in mouse naive mESCs (KLF5 and TFCP2l1); however, several seem unique to humans (e.g., TFAP2C), and some critical regulators in mice (e.g., *Esrrb*) are not typically upregulated in human naïve cells (Kisa et al., 2017; Rostovskaya et al., 2019). Consequently, there are substantial differences in transcriptional regulation in humans (Table 2), and the full naïve and primed regulatory networks remain to be elucidated.

**TABLE 2:** Epigenetic factors implicated in naïve and primed hESC control.

Gene name	Function	Effect on naïve or primed hESCs	Epigenetic pathway	References
*EZH2*	Histone methyltransferase	Required to primed hESCs, but dispensable in naïve hESCs	H3K27me3	Shan et al., 2017
*HDAC1/3*	Histone deacetylase	Histone deacetylase inhibition was required to establish naïve-like hESCs	Histone deacetylation/transcriptional regulation	Ware et al., 2014
*MCRS1*, *TET1*, *THAP11*	DNA demethylation (TET1) and epigenetic remodeling	Expressed in combination and can convert primed hESCs to naïve-like cells	5mC to 5hmC conversion (TET1) and unknown	Durruthy-Durruthy et al., 2016
*NNMT*	Nicotinamide N-methyltransferase	Knockout in primed hESCs leads to cells to acquire some naïve characteristics	Reduces histone methylation by removing the methyl-group donor SAM	Sperber et al., 2015
*SIRT2*	Histone/protein deacetylase	SIRT2 controls primed hESC state	Acetylation and regulation of glycolytic enzymes	Cha et al., 2017
*TNKS1/2*	Chromatin remodeling	Tankyrase 1/2 inhibition promotes naïve and extended pluripotency	Telomere elongation	Zimmerlin and Zambidis, 2020
*TET1/2*	DNA demethylation	Required for pluripotency in primed but not the naïve state	5mC to 5hmC conversion	Finley et al., 2018

*hESC, human embryonic stem cell; SAM, *S*-adenosylmethionine.*

### Epigenetic Influence of the Chemical Cocktails That Convert Primed to Naïve Cells

The human naïve cocktails begin with 2iLIF as a starting base, although LIF can be substituted for other molecules, and LIF is not strictly required in mouse naïve cells. Beyond 2i, many other inhibitors and signaling factors have been used, targeting JNK, MAPK, BRAF, SRC, and ROCK kinases. These inhibitors likely have widespread downstream effects on epigenetic regulation, although the pathways have not been fully explored. The two small molecules that have been directly implicated in epigenetic control are HDAC inhibitors, which are useful as a pretreatment of primed cells before conversion to naïve cells (Ware et al., 2014), and vitamin C. Vitamin C acts as a co-factor for TET and Jumonji (JMJ) domain-containing proteins. JMJ domain proteins are involved in histone demethylations, while TET domains convert 5mC to 5hmC, which is the initial step in the DNA demethylation pathway (Teslaa and Teitell, 2015). Overexpression of *TET1*, along with *MCRS1* and *THAP11*, can drive cells toward a naïve-like state (Durruthy-Durruthy et al., 2016), indicating that TET1 is important in naïve cells. Nonetheless, how vitamin C modulates TETs and JMJs to promote the formation of naïve hESCs remains unclear. Other epigenetic pathways involved include the PRC2 component EZH2, which is required to maintain primed hESCs but is dispensable for naïve cells (Shan et al., 2017). As EZH2 is a key catalytic enzyme for the repressive histone mark H3K27me3, it suggests that human naïve cells may have reduced epigenetic repression, the same as mice, although, just as in mice, human naïve cells have higher overall levels of H3K27me3 (De Clerck et al., 2019). Possibly, the situation is similar to that of the mice, and H3K27me3 is lost at the promoters of critical genes, but overall H3K27me3 is elevated in response to reduced DNA methylation.

Ultimately, there remains argument over which of these naïve cocktails captures most of the naïve state. This led to an expansion of the model that the naïve and primed cells exist on a spectrum (Weinberger et al., 2016; Smith, 2017; Yang et al., 2019b) and led to ideas of multiple interchangeable naïve and primed modules that can be switched on or off under certain conditions (Figure 1; Theunissen et al., 2016; Cornacchia et al., 2019). This helps explain the differences in the naïve cocktails, as each cocktail can switch on certain modules but may fail to activate them all.

### Extra/Expanded-Capability Cells With Totipotent-Like Properties

An extra complication for the naïve and primed model is the existence of “extra-capability cells” that describe ESC-like cells that are pluripotent and also have some aspects of totipotency (Yang et al., 2017a, b; Gao et al., 2019). These cells are grown under culture conditions similar to naïve cells, but they drop the MEK inhibitor from 2i and include WNT pathway inhibitors and then either SRC and tankyrase inhibitors (EPSCs) or ROCK inhibitor (EPSs). First described in mice, EPS/EPSCs (extra/expanded pluripotent stem cells) can contribute to the trophoblast, which is a property that both naïve and primed mESCs and hESCs lack. However, the gold standard test of totipotency, the derivation of a complete mouse using only these cells, has not yet been reported. This suggests that their totipotent properties remain incomplete or that they lack full totipotency. Indeed, there is argument that while mouse EPS/EPSCs can occasionally localize in the trophoblast, these cells lack trophoblast-specific markers, still express epiblast markers, and do not have totipotent properties (Posfai et al., 2021). Additionally, the DNA methylation state of EPS/EPSCs is somewhat contradictory. EPSCs have high levels of methylated DNA (Yang et al., 2017a), which does not match the DNA hypomethylation of totipotent embryonic cells and 2iLIF-grown naïve mESCs, although it should be noted that it is unclear if the hypomethylation in 2iLIF cells is a cell type effect, or is a side effect of PD0325901, a component of the 2iLIF cocktail. One advantage that the EPS/EPSCs do have is in the derivation of cells from species that have been previously resistant to the isolation of ESCs, for example, deriving porcine EPSCs (Gao et al., 2019). The EPS and EPSC cocktails have also been applied to human embryos to derive putative totipotent cells (Yang et al., 2017b; Gao et al., 2019). Overall, the identity of the EPS and EPSCs remains unclear, particularly how they correspond to *in vivo* development. EPS and EPSCs may hint at a further expansion to the module concept, where EPS and EPSCs are switching off some naïve modules and activating some totipotent modules. But like the naïve/primed split, these cell types are potentially activating only some of the totipotent modules, and only partial totipotency is achieved. A fascinating study of how biological phenotypes can act independently is the reprogramming of mESCs to oocyte-like cells (Hamazaki et al., 2020). In that study, oocyte-like cells could be derived without PGC specification, meiosis, or epigenetic reprogramming of DNA demethylation. This suggests that these aspects are independent and can be switched on and off in a module-specific fashion (Hamazaki et al., 2020).

### Links Between Metabolism and Epigenetic Control of the Naïve and Primed States

Pluripotent stem cell fate transitions from naïve to primed are accompanied by a metabolic switch, from oxidative phosphorylation (oxphos) to mainly glycolysis, respectively (Figure 1; Teslaa and Teitell, 2015). This mirrors the developing embryo, which mainly uses pyruvate and oxphos from fertilization to the blastocyst stage, before transitioning to glucose-based glycolysis and anaerobic metabolism in the late blastocyst (Devreker and Englert, 2000; Chason et al., 2011). Although it should be noted that it is unclear if glycolysis is required to produce energy for embryos, instead, it may be needed for biosynthetic pathways (Smith and Sturmey, 2013). ESCs, conversely, are highly active cells that divide rapidly and need a lot of energy. Hence, the link between embryonic metabolism and ESC metabolism is not a complete match. There is nonetheless a close link between metabolism and epigenetic control that has not been thoroughly explored (Shyh-Chang and Ng, 2017). For example, *SIRT1* is high in hESCs and acetylates and activates glycolytic enzymes (Cha et al., 2017), and in mice, HIF1A controls glycolytic/oxphos metabolism and influences cell state (Zhou et al., 2012). Many of the reactants required for epigenetic control are metabolic products. For example, acetyl-CoA is the main acetyl donor for histone acetylation, and intracellular levels of acetyl-CoA directly regulate global histone acetylation, and so function as a signal for overall cellular energy metabolism (Cai et al., 2011). An analysis of naïve and primed hESC states revealed that naïve cells express nicotinamide N-methyltransferase (*NNMT*) at high levels (Sperber et al., 2015), which is responsible for metabolizing *S*-adenosylmethionine (SAM), the major chemical donor for histone methyltransferases. As a consequence, naïve cells have low levels of SAM and correspondingly low levels of histone methylation, while primed cells have the inverse, high SAM and high histone methylation (Sperber et al., 2015). Importantly, these metabolic changes may be required for the naïve to primed transition, as *NNMT* knockout cells transition toward a primed state (Sperber et al., 2015). Similarly, 2iLIF-grown mESCs maintain high levels alpha-ketoglutarate that biases the cells toward DNA and histone demethylation by promoting the activity of JMJ-containing demethylases (Carey et al., 2015). In addition to these direct links between metabolism and chromatin, manipulation of metabolism by altering the growth medium also has strong effects on hESCs. Lipid deprivation of primed hESCs reverts them to an intermediate naïve-like state, and the reapplication of lipids pushes the cells toward a primed state (Cornacchia et al., 2019). The exact metabolic/epigenetic pathways behind this effect remain to be elucidated, but lipid deprivation may promote glucose utilization by glycolysis, making more acetyl-CoA available and leading to increased histone acetylation and gene activation. Similarly, mouse 2iLIF-grown cells can utilize fatty acid oxidation (FAO); and inhibition of FAO leads to a reversible quiescence (Khoa et al., 2020), reminiscent of diapause in mice where embryos can be paused if the developmental environment is unfavorable. This effect is driven by the activity of MOF, a histone acetyltransferase that acetylates histones at FAO-related genes and helps activate them (Khoa et al., 2020). Ultimately, there is an intimate interdependence between metabolism, and epigenetic control in embryonic cells and the embryo (Chason et al., 2011), which remains to be comprehensively explored (Betschinger, 2017).

### Epigenetic Control of the X Chromosomes in Female Cells

The epigenetic status of the X chromosomes in female ESCs is a particularly important point when discussing human naïve cells and is considered something of a hallmark for the naïve state (Nichols and Smith, 2009; Theunissen et al., 2016). In mice, the situation is relatively straightforward; in naïve mESCs, both X chromosomes are active; *Xist*, a long non-coding RNA that silences one X chromosome is not expressed; and in primed EpiSCs, *Xist* is expressed and one X chromosome is epigenetically inactivated (Bao et al., 2009). This roughly matches the *in vivo* embryonic states: in the late inner cell mass, both X chromosomes are active, and during differentiation in the late epiblast, one random X chromosome is silenced (Okamoto et al., 2004, 2011). The situation in human cells is more complex. Female hESCs (i.e., primed state) have one active and one inactive X chromosomes, which is strong evidence that hESCs are developmentally later than the early blastocyst (Nichols and Smith, 2009). Human naïve cells, depending upon the protocol used, have varying states of X chromosome inactivation, including intermediate states of *XIST* expression and epigenetic silencing (Sahakyan et al., 2017). The discrepancy between mice and humans may, at least partly, be related to the mechanism of X chromosome inactivation in humans, which appears to be more complex than in mice (Patrat et al., 2020). Briefly, in humans, *XIST* is expressed in the inner cell mass, but its expression is not correlated with epigenetic suppression (Okamoto et al., 2011; Petropoulos et al., 2016). Instead, one X chromosome is “dampened” by an unclear mechanism, before later full X chromosome inactivation by chromatin silencing (Petropoulos et al., 2016), possibly as late as post-implantation. This added complexity in humans may explain the differences in X chromosome status in naïve and primed hESCs or may reflect species-specific epigenetic regulatory differences (Okamoto et al., 2011).

Ultimately, human naïve ESCs remain surprisingly elusive to pin down (Theunissen et al., 2016). A close comparison of gene expression profiles and epigenetic states indicates that naïve hESC protocols, to date, remain distinct from naïve mESCs (Yang et al., 2019b), suggesting that the current protocols only capture aspects of the naïve state. As the conversion of primed to naïve cells is an artificial conversion, not only are potent epigenetic barriers in place, but the route to true naïve cells is unclear (Figure 2). Another, perhaps uncomfortable, possibility is that the naïve mouse state has no clear mimic in humans, is species-specific, or is a transitory stage in humans that cannot be stably captured *in vitro* (Rossant and Tam, 2017; Yang et al., 2019b).

## Epigenetic Control in Reprogramming Somatic Cells to Pluripotent Stem Cells

Interconversions between closely related embryonic states have helped inform our understanding of the epigenetic control of embryogenesis. Another, more drastic, cell conversion is the reprogramming of somatic cells to induced pluripotent stem cells (iPSCs) or by SCNT (Dean et al., 2003; Takahashi and Yamanaka, 2006). These two techniques have revolutionized the study of epigenetic regulation of the embryonic state, particularly in humans where early embryogenesis is hard to study. Both reprogramming techniques involve the global reconfiguration of gene expression patterns driven by epigenetic remodeling (Liu et al., 2020), and these methods have revealed potent epigenetic barriers that restrain cell type conversions (Xu et al., 2016). Chromatin is dramatically reorganized during reprogramming (Wang et al., 2017), as enhancer–promoter interactions and active and repressive sequences make new contacts and reorder the transcriptional program (Apostolou et al., 2013; Di Stefano et al., 2020; Lu et al., 2020b). During somatic cell reprogramming, the ectopic pluripotency transgenes *Oct4*, *Sox2*, *Klf4*, and *Myc* reconnect target enhancers to promoters to induce transcriptional change (Wei et al., 2013; Beagan et al., 2016; Stadhouders et al., 2018). More importantly, during this process, chromatin reorganization occurs prior to, or independent from, gene expression changes (Wei et al., 2013; Beagan et al., 2016). Indeed, changes in chromatin accessibility often precede changes in gene expression, often by several days (Li et al., 2017a).

**FIGURE 3:**
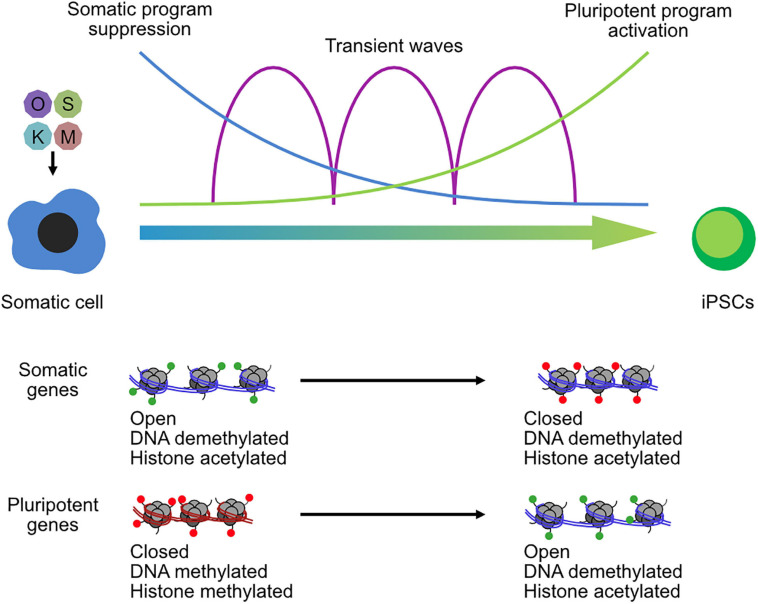
Chromatin reconfiguration in the reprogramming of somatic cells to induced pluripotent stem cells. OSKM transgenes are transfected into somatic cells and initiate a complex series of biological programs, including the suppression of the somatic program in the early phase of reprogramming and the activation of the pluripotent program in the late phase. Transient waves of gene expression programs occur between the two-cell type states. Chromatin is remodeled throughout the reprogramming process, and especially at somatic loci and pluripotent gene loci that open and close chromatin and lose or gain histone methylation.

Broadly, somatic cells tend to have higher levels of repressive marks, which are reduced during the reprogramming to pluripotency. Vitamin C improves the reprogramming of somatic cells to pluripotency by modulating TET and JMJ domain-containing proteins (Wang et al., 2011; Chen et al., 2013a), leading to demethylation of DNA and H3K36me2/3. Other repressive epigenetic marks have been identified as major barriers for reprogramming (Arabaci et al., 2020), particularly the repressive histone modification H3K9me3 that is redistributed during iPSC reprogramming (Hawkins et al., 2010). Methyltransferases are downstream targets of BMPs and are a determinant for iPSC generation by regulating the methylation states at core pluripotency loci (Chen et al., 2013b). Similarly, the loss of the H3K9 methyltransferase *Setdb1*, or its co-factor *Trim28*, leads to improved reprogramming (Miles et al., 2017), although it may ultimately be deleterious as it causes spontaneous differentiation in the resulting iPSCs (Klimczak et al., 2017). Other H3K9me3 enzymes also impair the conversion of somatic cells to iPSCs, including *Suv39h1*/*2* and *Ehmt2* (G9a), along with the H3K79me3 methyltransferase *Dot1l* (Onder et al., 2012). However, the regulation of repressive histones is more subtle than just repressive mechanisms are bad for reprogramming. Reprogramming is a multi-phased program (Brambrink et al., 2008), and one of the earliest phases is the large-scale suppression of the somatic gene expression program (Chronis et al., 2017; Li et al., 2017a). Whether an epigenetic enzyme is beneficial or deleterious for reprogramming may ultimately depend upon the balance of its role in suppression or activation of the somatic and pluripotent programs (Figure 3). Outsize roles in suppression of the somatic or activation of the pluripotent program are likely to improve reprogramming, while the opposite is likely to shift the balance toward impairment. Consequently, epigenetic regulators have context-specific and temporal-specific effects during reprogramming. For example, knockdown of the co-repressors *Ncor1*/*Ncor2* is deleterious for the early stages of reprogramming, due to reduced somatic gene suppression, but beneficial for the late stages due to reduced pluripotent gene repression (Zhuang et al., 2018). A similar pattern was observed for the histone H3K27 demethylase *Kdm6b* (JMJD3) (Huang et al., 2020) and the H3K27me3 methyltransferase *Ezh2* (Rao et al., 2015). Other epigenetic regulators can be beneficial in both phases, although they may use different mechanisms to achieve this effect. For example, the H2AK119 ubiquitinase RYBP cooperates with PRC1 complex to suppress the somatic program via the histone demethylase KDM2B but cooperates with OCT4 to activate the pluripotent program (Li et al., 2017b). Epigenetic regulators can be something of a double-edged sword for somatic cell reprogramming (Onder et al., 2012; Li et al., 2017b; Zhuang et al., 2018).

A curious observation in studies of epigenetic reprogramming is that the loss of epigenetic regulators can have strong effects on the reprogramming process (Xu et al., 2016), yet the loss of the same factors in ESCs tends to have a much weaker or negligible effect. For example, knockdown of *Ncor1/2* improves reprogramming but does not affect mESCs (Zhuang et al., 2018). Similarly, *Sin3a/Sap30* loss impairs reprogramming (Li et al., 2017a; Saunders et al., 2017) but causes no change in mESCs. The short-term knockdown of a panel of 40 epigenetic regulators resulted in differentiation in only two knockdowns (He et al., 2019). These observations indicate that epigenetic pathways involved in the establishment of pluripotency may not always be involved in the maintenance of pluripotency or, if they are involved, can often be in contradictory ways. A good example is the knockdown of the PRC2 component *Ezh2*. Knockdown promotes reprogramming (Onder et al., 2012), but in mESCs, it affects self-renewal and makes the cells prone to differentiation. Overall, this suggests two patterns for epigenetic regulators in reprogramming and pluripotent maintenance: (1) once the reprogramming epigenetic barriers have been overcome, they are dispensable in ESCs, and (2) loss of epigenetic regulators makes ESCs unstable and more prone to differentiation. Epigenetic barriers are not always two-way and often act more like valves that can be easily traversed again if going in the opposite direction. This effect puts a limitation on screening technologies such as genome-wide knockdowns/outs and sgRNA screens. Epigenetic factors that impact the stable ESC state may not be relevant to the entirety of the reprogramming process. Consequently, challenging experiments must be performed during the reprogramming time course to understand the requirements for the temporal order of events. So far, these screens have focused on the early stages of reprogramming, which is more experimentally tractable (Borkent et al., 2016; Toh et al., 2016; Miles et al., 2017; Neganova et al., 2019). However, reprogramming is a curiously multistage-phased process, and epigenetic barriers may be transiently erected and disassembled, meaning that screening also needs to be timed to specific stages. Potentially, technologies such as Perturb-seq (Dixit et al., 2016), which provides candidate factors and phenotype readout simultaneously, may help in understanding the full range of epigenetic barriers blocking reprogramming.

## Transposable Elements, the Early Embryo, and NaÏVe and Primed Embryonic States

Transposable elements are the single largest constituent of mammalian genomes (Hutchins and Pei, 2015), taking up around 40% of the total DNA sequence. They can be divided into four broad categories, DNA transposons, and three types of retrotransposon: long-interspersed elements (LINEs), short interspersed elements (SINEs), and the endogenous retroviruses/long-terminal repeats (ERVs/LTRs). TEs have been viewed as genetic parasites that are especially dangerous during embryogenesis when transposition duplications are capable of entering the germline. However, TEs can act as a source of evolutionary innovation, by duplicating TF binding sites, rewiring gene regulatory networks, altering splicing patterns, and many other effects on the genome and cell (Bourque et al., 2018), both beneficial and deleterious (Enriquez-Gasca et al., 2020). During early embryonic development, the genome is reprogrammed back to a naïve state. This process involves the global DNA demethylation of the genome, a process that is presumed to be a requirement for the correct execution of a new developmental program. However, DNA methylation is also one of the dominant mechanisms for the suppression of TEs in somatic tissues (Feng et al., 2010; Jonsson et al., 2019), and global DNA demethylation in the early embryo helps release waves of TE expression (Beraldi et al., 2006; Goke et al., 2015). Intriguingly, TE activity is dynamic in early embryonic cells (Wang et al., 2020) and is both stage and TE type-specific (Goke et al., 2015). In the early embryo and ESCs, instead of DNA methylation suppressing TE expression, other mechanisms take over, particularly the methylation of histone H3K9me3 by SETDB1, which is recruited to specific TEs by TRIM28 binding to KRAB-family zinc finger TFs (Ecco et al., 2016). However, there is also evidence that a wide range of epigenetic enzymes are involved in the suppression (or management) of the expression of TEs. Indeed, the early embryo can contain vast quantities of TE RNAs, a single MaLR LTR family TE can comprise up to 13% of the total oocyte RNA (Peaston et al., 2004), and SINE elements may make up a further 3% (Bachvarova, 1988). Functional roles for TEs in embryogenesis are less well explored, but LINE L1 expression is required for progression to the blastocyst stage (Percharde et al., 2018), while depletion of L1s in mESCs leads to the derepression of genes that are proximal to LINE L1s (Lu et al., 2020a). HERVKs are expressed and produce viroid-like particles in normal human embryos (Grow et al., 2015). Multiple lines of evidence indicate that TE expression and epigenetic activity, aside from retrotransposition, are involved in the embryonic process, although their full involvement, both beneficial and deleterious, is unclear.

### Transposable Elements and Two-Cell-Like Cells

**FIGURE 4:**
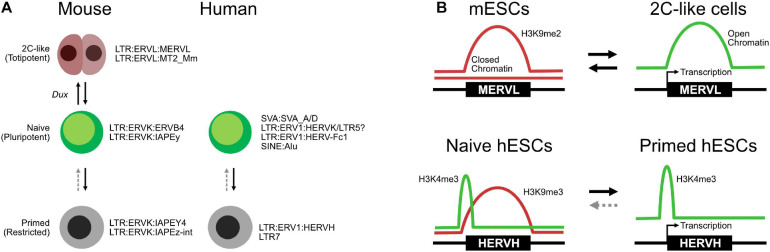
Transposable elements (TEs) in totipotent, naïve, and primed embryonic states. **(A)** Expression of specific TEs mark cell type states in both mice and humans, although the TEs involved are species-specific. **(B)** Selected chromatin transitions at specific MERVLs in mouse embryonic stem cells (mESCs) during the transition to two-cell (2C)-like cells and at HERVHs in naïve and primed human ESCs (hESCs).

TE expression has found utility as both a marker of embryonic stages and also as a tool to isolate new cell types with enhanced features. In mESC cultures, there is a small subpopulation (about 1%) that expresses a mouse-specific MERVL ERV (Macfarlan et al., 2012). Intriguingly, MERVL is expressed in the 2C stage of the mouse embryo when the cells are still totipotent. Isolation of the MERVL expressing “2C-like” cells from an mESC culture resulted in a population of cells that cycle into and out of the mESC state and have some totipotent-like properties. For example, they can partially colonize the embryonic trophoblast, a capability that normal mESCs lack. The 2C-like cells are transient and cannot be maintained, but various cocktails and protocols have been developed that improve their derivation (Iturbide and Torres-Padilla, 2020). Mechanistically, 2C-like cells rely on a transcriptional network distinct from the OCT4–SOX2 pluripotency network. The details are still being worked out, but the 2C-like state centers around several families of TFs and microRNAs, including miR344, DPPA2/4, ZSCAN4-family, ZMYM2, NELFA, and GATA2 (De Iaco et al., 2019; Fu et al., 2019; Zhang et al., 2019; Hu et al., 2020; Yang et al., 2020). These pathways ultimately center on the expression of *Dux* (Figure 4A; Percharde et al., 2018), a key TF required for ZGA in the developing embryo (De Iaco et al., 2017). This 2C-like transcriptional network cooperates to remodel the epigenetic state, H3K9me3 and H2AK119ub1 (ubiquitination), and chromatin assembly by CAF1, which are all particularly critical (Ishiuchi et al., 2015). MERVLs themselves tend to lack H3K9me3 and are not bound by SETDB1 (Maksakova et al., 2013) but are marked by H3K9me2 and H3K56ac (Macfarlan et al., 2011; He et al., 2019). Knockdown of *Ehmt2* (G9a) leads to the upregulation of MERVLs by a direct mechanism involving loss of H3K9me2 at MERVLs and the gain of open accessible chromatin (Figure 4B; Maksakova et al., 2013; Hendrickson et al., 2017). Similarly, *Kdm1a*, a histone demethylase, is important for suppressing the expression of MERVLs (Macfarlan et al., 2011), although the exact mechanism by which KDM1A suppresses MERVLs is not clear, as KDM1A can demethylate both H3K4me1 and H3K9me2. H3K4me1 marks enhancers and is generally associated with gene activation, while H3K9me2 is a repressive mark, often associated with heterochromatin and H3K9me3. H3K9me2 marks MERVLs, but H3K4me1 does not, suggesting that KDM1A may at least partially regulate MERVLs indirectly. Indeed, several direct and indirect chromatin modifiers regulate MERVLs. Both TRIM28 and RNF2 do not bind directly to MERVLs, and their corresponding marks, H3K9me3 and H2AK119ub, are not found either (Maksakova et al., 2013; He et al., 2019), but knockdown of *Trim28* or *Rnf2* leads to upregulation of MERVLs. Curiously, histone ubiquitination has two roles in both suppressing and activating MERVLs: knockdown of the histone H2A ubiquitinase *Rnf2* leads to the deubiquitination and upregulation of MERVLs (Zhang et al., 2019), while conversely, knockdown of the H2B deubiquitinase *Usp7* leads to H2B ubiquitination and upregulation of MERVLs (Chen et al., 2020). There is a similar pattern here to the conversion of primed cells to naïve cells: generally, the loss of repressive histone marks is beneficial for the conversion of cells to a 2C-like state. For example, knockdown of *Setdb1* (Wu et al., 2020), *Trim28* (KAP1) (Maksakova et al., 2013), *Dnmt1* (Fu et al., 2019), and *Rnf2* (RING1B) (He et al., 2019; Zhang et al., 2019) and inhibition of HDACs by trichostatin A (TSA) (Macfarlan et al., 2012) can all increase the number of 2C-like cells in an mESC culture.

TE activity, both as transcribed RNAs and as enhancers, is linked with the 2C-like state. In oocytes, a key factor is DPPA3 (*Dppa3*/*Stella*), a maternally inherited protein that is essential for the transition from the maternal to the zygotic gene expression program (Huang et al., 2017). When *Dppa3/*DPPA3 was removed from the maternal pool, MERVLs failed to be upregulated, and the embryos arrested at the 2C stage (Huang et al., 2017). Intriguingly, the authors found that microinjection of small interfering RNAs (siRNAs) targeting MERVLs led to a reduction in MERVL-derived Gag proteins and developmental impairment (Huang et al., 2017). These results suggest that MERVL expression is not simply a marker for the 2C stage but is also functionally relevant. MERVL sequences are spliced into other transcripts as TE–gene chimeras (Huang et al., 2017; Chen et al., 2020), and MERVL expression may also be driving transcript expression. MERVL sequences can act as an enhancer to recruit TFs to promote transcription (Huang et al., 2017; Zhang et al., 2019), and the MERVL sequence can act as a promoter (Jiang et al., 2020). In addition to MERVLs, LINE L1 RNAs silence *Dux* expression by recruiting TRIM28 to induce heterochromatin via H3K9me3 (Percharde et al., 2018). Knockdown of LINE L1 RNAs leads to reactivation of the 2C-like gene expression program and particularly reactivation of Dux (Percharde et al., 2018). This points to a surprisingly complex relationship between TE expression, transcriptional regulation, and epigenetic control of heterochromatin. LINE L1 RNAs can reactivate *Dux*, which then appears to lead to deregulation of H3K9me3, which activates MERVLs, which are spliced into key 2C-like transcripts and may also act as enhancers for genes required for the 2C-like state. Ultimately, the causal relationship between TE activation, 2C-like gene expression programs, and transcriptional and epigenetic control still needs to be unpicked, but it is a fascinating model system for the establishment of totipotency.

### Transposable Elements and Human Naïve Cells

The expression of TEs has also found utility as markers of the embryonic state in hESCs (Theunissen et al., 2016). hESCs express the primate-specific HERVH ERV, and their accompanying LTR (LTR7) can act as pluripotent-specific transcription start sites (Fort et al., 2014). This pattern of TE expression has been proposed as one of several criteria that define the naïve and primed hESC states (Theunissen et al., 2016). Briefly, primed hESCs express HERVH/LTR7 RNAs, while naïve cells express a more mixed set of TEs, but particularly SVAs, LTR5, and HERVK. HERVH are marked by H3K4me3 in both naïve and primed cells but are typically marked by the repressive H3K9me3 mark in primed cells (Figure 4B; Theunissen et al., 2016), although another study suggests that high levels of HERVH specifically mark naïve hESCs (Figure 4A; Wang et al., 2014). An interesting observation of that study was the splicing of HERVH directly into hESC chimeric transcripts. This is similar to the splicing of MERVLs seen in 2C-like cells, suggesting that a robust understanding of TE splicing patterns may lead to insights into embryonic cell states.

Transposable elements can be expressed as fragmentary RNA, and an area that remains poorly explored is the splicing of TEs into other transcripts. TEs can be expressed as individual units within the cell, but they can be spliced into longer transcripts, often as part of long non-coding transcripts but also into normal coding transcripts to generate novel chimeric transcripts (Bourque et al., 2018). To date, exploring the contribution of TEs to the normal transcriptome of a cell has been hampered by the use of short-reads to assemble transcripts (Babarinde et al., 2019). Nonetheless, the chimeric splicing of TEs into transcripts is a feature of pre-implantation embryonic cell types. The mouse-specific MERVLs that are transcribed in 2C-like cells are spliced into other coding and non-coding transcripts (Macfarlan et al., 2012; Huang et al., 2017; He et al., 2019). Similarly, the HERVH human-specific ERVs that are a feature of pluripotent stem cells are also spliced into other transcripts (Fort et al., 2014; Wang et al., 2014). Intriguingly, TEs are spliced into pluripotent transcripts in cancerous cells (Jang et al., 2019), the implication being that these TEs are activating pluripotent genes and converting them to oncogenes. However, HERVH activation appears not to be a general feature of cancer (Zapatka et al., 2020).

When TEs are still retrotranspositionally active, it poses a danger to the cell; however, once the coding sequences are mutated, and they are no longer functional, and epigenetic suppression mechanisms should decline due to a lack of evolutionary pressure to suppress TEs. Yet TEs maintain complex epigenetic regulatory patterns that are TE-type specific and are present long after they have stopped being capable of retrotransposition and are several million years old (Bourque et al., 2008; He et al., 2019). This suggests regulatory function and co-option for legitimate biological function. A good example is H3K9me3, a critical epigenetic mark responsible for silencing TEs in mESCs (Rowe et al., 2013; Yang et al., 2015), which is intimately involved in 2C-like cells, naïve cells, and reprogramming (Chen et al., 2013b; Bao et al., 2015; Xiao et al., 2017; Wang et al., 2018; Wu et al., 2020). H3K9me3 is remodeled during embryonic development, particularly at LTRs and ERVs (Wang et al., 2018). Knockdown of several H3K9me3-related factors, *Setdb1* and *Trim28*, impaired mouse embryonic development to the blastocyst, but *Chaf1a* (a modulator of H3K9me3 and part of the CAF1 complex) knockdown nearly completely blocked embryos from progressing past the morula stage (Wang et al., 2018). Consequently, H3K9me3 seems to be performing double duty as a major repressive mark for LTRs and ERVs, as well as erecting epigenetic barriers between cell type conversions. Ultimately, there is a tight integration between epigenetic control of TE activity and cell fate, and they should be considered as a unified mechanism with overlapping activities.

## Conclusion

Epigenetic reconfiguration during early embryonic development is a critical process that resets the cells and makes them capable of a new round of development. The epigenetic rearrangements on chromatin are widespread and encompass changes in histone modifications, nucleosome positioning, 3D structure, and DNA modifications. A complex system of epigenetic regulators is involved in this process, and there are many distinct stages that cells transition through during normal development. Some of these states can be captured *in vitro* and have informed our understanding of the mechanisms behind embryonic development, and how autonomous and exogenous signaling, transcriptional control, and epigenetics combine to regulate development. Many mysteries remain, particularly in the role of epigenetic control in maintaining and blocking cell type conversions. Understanding this process in detail will lead to an enhanced understanding of cell type transitions that will inform potential medical treatments, particularly cell replacement therapy.

## Author Contributions

LS, XF, and GM compiled the tables and prepared the figures. All authors were involved in manuscript writing. AH wrote and approved the final text, and funded the study.

## Conflict of Interest

The authors declare that the research was conducted in the absence of any commercial or financial relationships that could be construed as a potential conflict of interest.
